# Coronary Revascularization of Giant Aneurysms in Children With Kawasaki Disease: A Report of Two Cases

**DOI:** 10.3389/fped.2020.547369

**Published:** 2020-09-18

**Authors:** Katsumi Akimoto, Mana Harada, Hisayuki Oda, Takeshi Furukawa, Ken Takahashi, Masahiko Kishiro, Toshiaki Shimizu, Keisuke Nakanishi, Shiori Kawasaki, Atsushi Amano

**Affiliations:** ^1^Pediatrics and Adolescent Medicine Department, The Juntendo University, Tokyo, Japan; ^2^Cardiovascular Surgery Department, The Juntendo University, Tokyo, Japan

**Keywords:** kawasaki disease, children, coronary artery bypass grafting, ischemic heart disease, collateral artery

## Abstract

In recent years, >100 cases of coronary artery stenotic lesions due to Kawasaki disease were treated with coronary artery bypass grafting (CABG). Surgical indications include stenosis of >75%, myocardial infarction history, electrocardiographic changes, and ischemia, as detected by myocardial scintigraphy and electrocardiography, due to drugs or exercise. Some centers have reported good patency rates, even in infants. The advantages of CABG in younger patients are minimal loss of left ventricular function, early elimination of post-operative ischemia risk, and improved quality of life. However, the disadvantage of performing CABG in younger patients is the small coronary artery diameter and the thin vessel wall, which can lead to post-operative occlusion, especially when performed by inexperienced surgeons. The optimal timing of CABG varies by institution and case, which depends on the presence or absence of complications, such as left ventricular dysfunction or valve regurgitation, and surgeon's experience. Importantly, unlike adult surgery, childhood CABG needs to be kept open for the very longest possible period of time to determine the optimal conditions for surgery. We report two pediatric cases of giant coronary artery aneurysms diagnosed in infancy. During school age, the patients had a mild decline of left ventricular ejection function. In one case, there were no clinical symptoms because of the development of collateral vessels and limitation of exercise. Both patients underwent surgery with good results. The gastric gastroepiploic artery could be anastomosed owing to the development of collateral blood vessels, although it was obstructed. At 1 year after surgery, both patients had a good post-operative course without complications of anastomotic stenosis or myocardial damage due to aneurysm resection. If conditions are favorable, bypass surgery can be postponed to several years until the coronary arteries are sufficiently large to warrant a delay in coronary artery stenosis in cases of infantile Kawasaki disease.

## Introduction

More than 40 years have passed since the first coronary artery bypass grafting (CABG) procedure in a child with Kawasaki disease (KD) was reported by Kitamura et al. (1). Improvements in treatment methods over time have reduced the number of cases involving giant coronary artery aneurysms to 0.2–0.3%. According to a nationwide survey conducted in Japan from 1999 to 2010, giant coronary artery aneurysm (CAL) rupture occurs within 1 month of KD onset, and there has been no death reported beyond 2 years (2). This suggests that treatment in the acute phase is important to avoid rupture, and the development of collateral circulation may help to avoid ischemic damage after the acute phase. In that survey, ~20 patients underwent CABG to treat coronary artery stenosis. The median time of CABG from disease onset was 56 months (range, 3–117 months). The optimal timing of surgery also depends on the individual patient's age, condition (including factors such as ischemia and collateral circulation), and the surgeon's experience level with the procedure.

In this report, we describe two pediatric cases in which their conditions prompted us to delay surgeries until the children reached school age. Specifically, the first case required the removal of a calcified giant aneurysm on the left anterior descending artery (LAD). The second case involved a stenosis distal to the right coronary artery (RCA) and required further growth of the right gastroepiploic artery (GEA). Both cases had adequate collateral circulation development and cardiac function preservation. CABG in children can be postponed until after infancy when the coronary artery diameter and wall thickness have grown. In addition, if the surgeon is inexperienced with performing CABG in children, we recommend waiting until the children weighed 15 kg to reduce the risk of complications and provide stable blood flow for a long period of time, even until adulthood.

## Case Presentation

### Case 1

The first patient was a 10 year-old boy (141 cm, 36.5 kg). KD was diagnosed at the age of 9 months. Two courses of high-dose γ-globulin therapy (IVIG) and steroid pulse therapy were performed. From disease day 13 to day 17, the proximal LAD dilated to 8 mm, and the proximal RCA dilated to 6 mm. The patient was then transferred to our hospital. IVIG therapy was administered again on the day of admission; however, a thrombus was detected in the LAD aneurysm detected on the 22nd pathological day.

He had no symptoms, but ST elevation was detected in the electrocardiogram (ECG) in leads II, III, aVF, and V3–V5. We diagnosed this case as acute myocardial infarction (AMI). Intracoronary thrombolysis (ICT) with tissue plasminogen activator (t-PA) was performed on the LAD while the patient was intubated, and sedation was performed with phenobarbital and midazolam. After ICT, heparin, and warfarin were administered to avoid thrombus formation, and verapamil was administered to avoid rupture in order to control spastic changes in the vessel and to maintain blood pressure <100 mmHg. Fever continued intermittently, and IVIG therapy was given on days 28 and 35. Warfarin was used to maintain the international normalized ratio (INR) at 2.0–2.5 and sedative therapy was continued until day 45 to maintain the patient at rest. The diameter of the aneurysm on proximal RCA was maintained at 6 mm, similar to the size observed during the acute phase. Although the thrombus disappeared, the left CAL grew, reaching 28 mm on day 57.

The patient was discharged on day 81 and therapy was continued with aspirin, angiotensin II receptor blockers (ARB), and warfarin. Stress ECG, chest radiography, echocardiography, and magnetic resonance coronary angiography (MRCA) were performed during outpatient visits to monitor for the development of stenosis or thrombus. After 3 months, his coronary angiography (CAG) revealed a giant CAL in the LAD and left circumflex artery (LCX).

At the age of 6 (22 kg), 5 years from disease onset, stenosis was found in the proximal part of the LCA aneurysm; however, no ischemic changes were seen on the treadmill test due to the development of collateral arteries from the RCA. He weighed 22 kg, and his coronary artery diameter was predicted to be well-developed, but the surgeon did not want to perform CABG because there were no ischemic findings and the large aneurysm adhered tightly to the myocardial tissue, it would be difficult to excise. Due to those findings, CABG was postponed.

**Figure 1:**
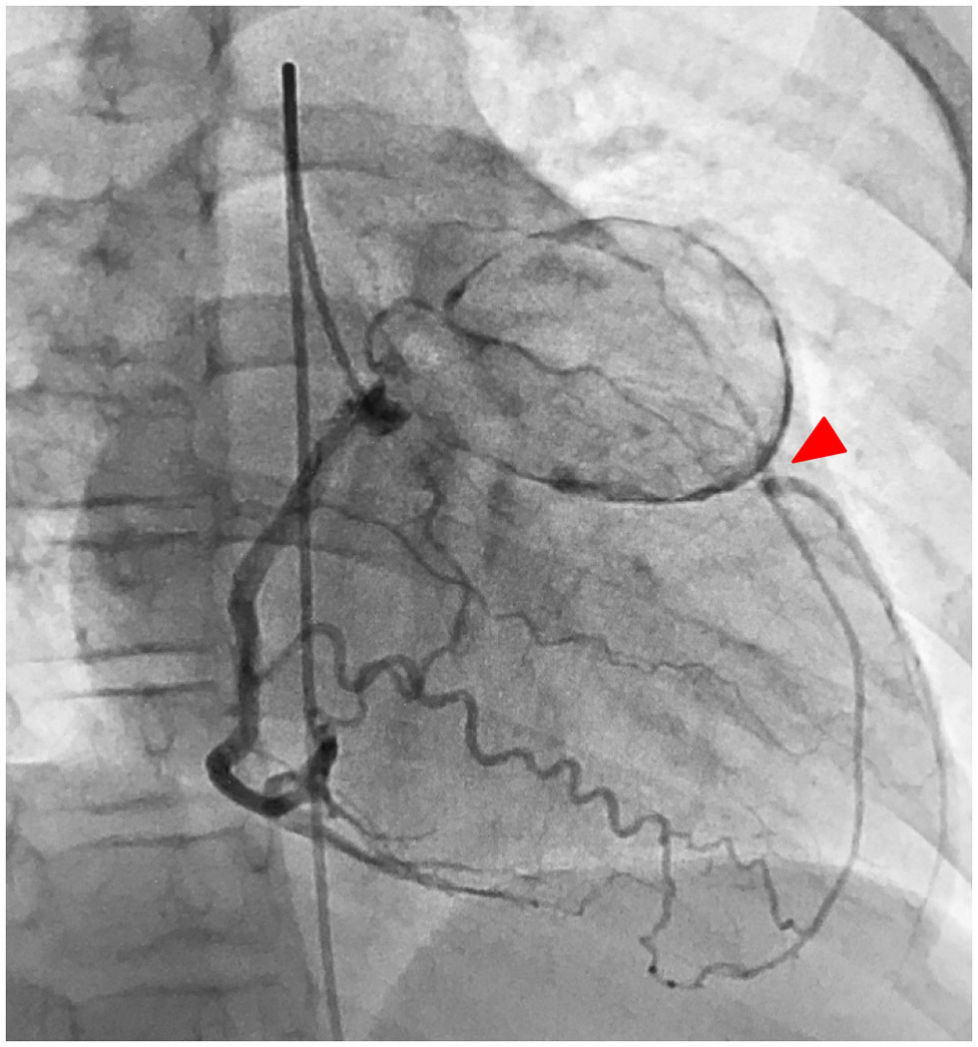
Case 1: left CAG showed a huge aneurysm with calcification of the proximal left coronary artery, and the LAD is obstructed at the outflow of the aneurysm (red arrowhead), and the blood is supplied by the collateral circulation tract from the right coronary artery. LAD, left anterior descending artery; LCX, left circumflex artery.

**Figure 2:**
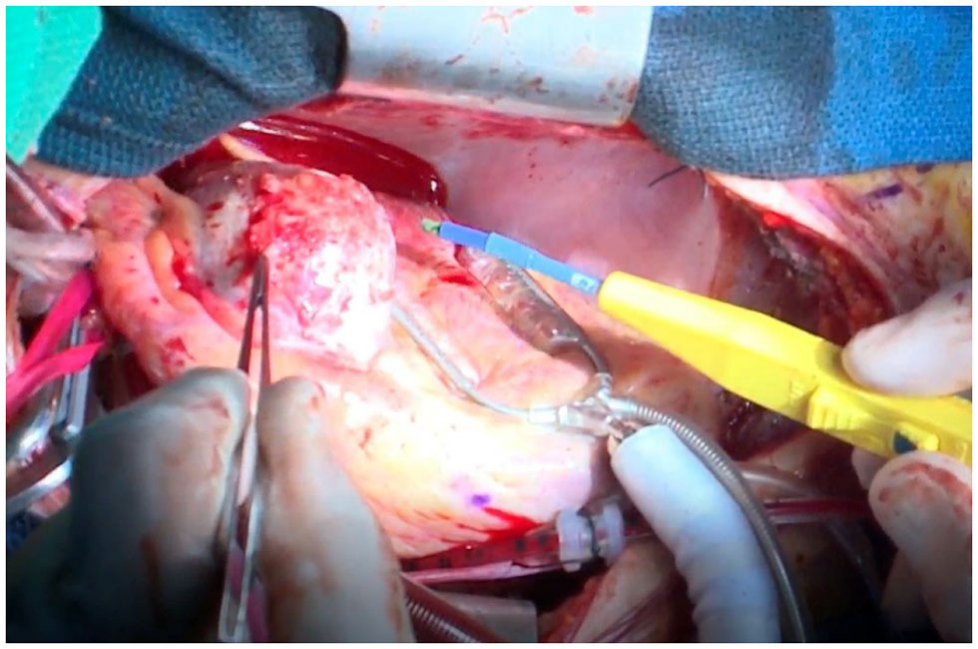
Case 1: macrofindings of the LAD aneurysm during surgery. A giant coronary aneurysm prior to resection is observed. A hard and calcified mass >40 mm in diameter is present. The mass was filled with a red blood clot. LAD, left anterior descending artery.

A treadmill test performed at 10 years of age, 8 years from disease onset, showed ST depression in the left chest lead during a regular check-up, and stress Tl myocardial scintigraphy showed extensive ischemia of the lateral wall; nevertheless, viability was maintained. CAG confirmed a huge aneurysm with calcification of the proximal left coronary artery and complete LAD occlusion at the outflow area of the aneurysm with collateral circulation from the RCA and stenosis of the left circumflex artery (LCX) (Figure 1). The RCA aneurysm disappeared completely. Left ventricular ejection fraction (EF) was 61%, indicating a decrease in sidewall contractions. Based on such findings, we believed that CABG was indicated. Surgery was performed using two arterial grafts: the left internal mammary artery (LITA)-LAD and the right internal mammary artery (RITA)–PL (posterolateral branch); the giant aneurysm (45 × 35 mm, severe calcification) was resected (Figure 2). The lumen of the giant calcified aneurysm was filled with a thrombus. The patient progressed well after surgery. Echocardiography at 1 year after the operation showed no changes in EF, which remained at 61%; however, coronary CT showed no stenosis or occlusion. Since the operation, the patient has been taking oral aspirin and carvedilol daily and living with exercise restrictions.

### Case 2

**Figure 3:**
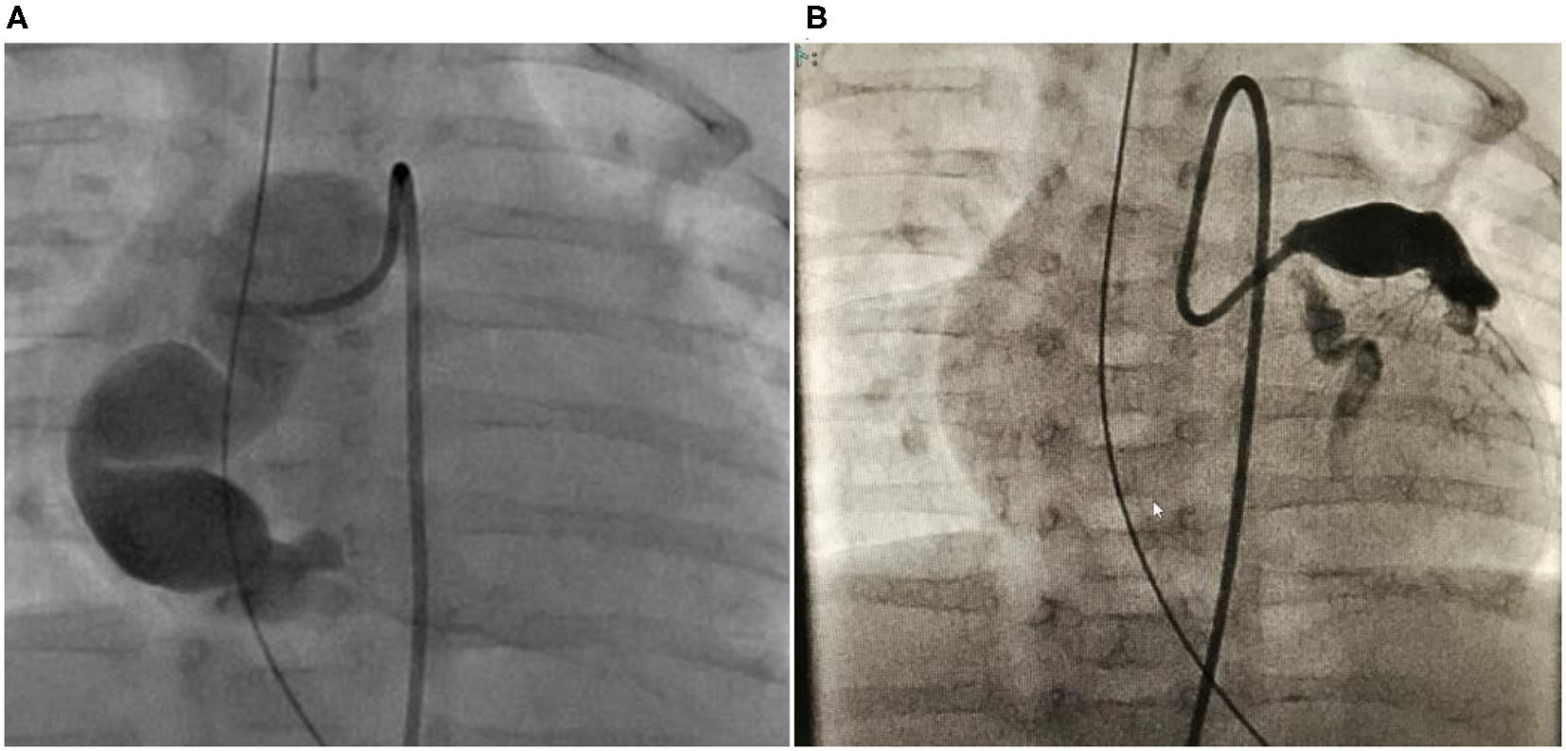
Case 2: right and left CAG performed in the acute phase when the patient was 2 months old. **(A)** It exhibited a large RCA “sausage-like” aneurysm at segments 1–3 with a maximum diameter of 12 mm. **(B)** Let CAG exhibited aneurysms in the LAD at segment 6 with a maximum diameter is 6 mm and LCX with a maximum diameter of 3.3 mm at segment 11. CAG, coronary angiogram; RCA, right coronary artery; LAD, left anterior descending artery; LCX, left circumflex artery.

The patient was a 7 year-old boy (107 cm, 16.4 kg). Disease onset occurred at the age of 2 months. Although IVIG was administered three times after IVIG and methylprednisolone pulse therapy, the inflammatory responses persisted, and bilateral coronary artery aneurysms were found 1 week after admission. The patient was transferred to our hospital on disease day 17. After admission to the intensive care unit, the patient was intubated to maintain sedation, and the heart rate and blood pressure were suppressed using nicardipine and sedatives. Despite those therapies, large aneurysms were detected at all three coronary arteries. At 3 months of age, 1 month from disease onset, there were no symptoms, but deepQ wave in lead III on ECG suggested ischemic changes and the troponin and CK-MB levels were elevated on blood test. We diagnosed the patient with AMI, and CAG was performed. On CAG, a huge long segmental aneurysm on RCA was observed with a maximum diameter of 12 mm, the blood flow was almost arrested, and the presence of a thrombus was confirmed (Figure 3). On the left side, the aneurysmal size on LAD was 9.2 mm and that on LCX was 3.3 mm.

Emergency ICT using t-PA was performed for the RCA thrombus. The thrombus disappeared on the echocardiogram; however, the fever persisted, and infliximab was administered on day 18. The body temperature began to decline on the next day, and the heart rate stabilized. One week later, the patient was extubated, and sedation was discontinued. Therapy was continued with aspirin, ARB, and warfarin. Thereafter, the patient underwent regular follow-up evaluations with ECG, chest radiography, echocardiography, and coronary angiography.

**Figure 4:**
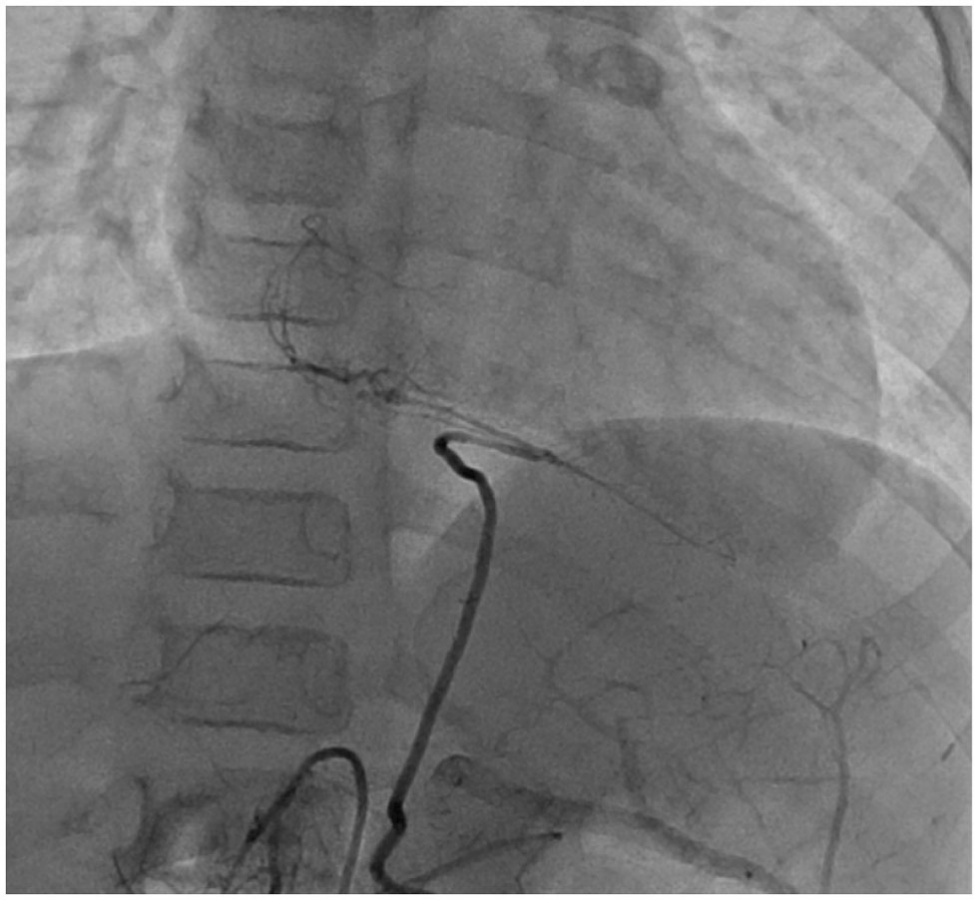
Case 2: right CAG at 1 year after CABG. The blood flow supply from the GEA to the distal site of RCA was adequate. CAG, coronary artery angiogram; CABG, coronary artery bypass graft surgery; GEA, gastroepiploic artery; RCA, right coronary artery.

At 1 year of age, 10 months after the acute phase, CAG revealed that the main RCA was completely occluded. However, peripheral blood flow was maintained from the LCX. The LAD vessel diameter still had aneurysmal change, but the diameter improved to 5 mm, no stenotic lesion was detected, and LCX also returned to its normal size. Subsequently, CAG exhibited similar findings as those observed at 1 year after the acute phase. At 2 years of age, 2 years from disease onset, CAG was performed and similar findings were observed. At that time, his body weight was <15 kg, and we speculated that performing CABG using RITA would be difficult at this age. Thus, CABG was postponed. After CAG, he went to the hospital for a regular check-up and underwent exercise ECG. He did not complain of any discomforts or chest pains, even while performing exercises at school. However, at 7 years of age (weight, 16.4 kg), 7 years from onset, he lost consciousness while exercising and underwent another CAG. The LAD was completely occluded and stenosis of the LCX of 0.9 mm was observed. Drug-loaded myocardial scintigraphy showed extensive ischemia of the sidewall; however, the left ventricular EF was maintained at 70%. He had symptoms and sufficient weight and ischemic findings on examination to warrant surgery. Then, CABG was performed. Anastomosis was performed at the following two places: LITA-LAD and RCA-GEA. However, the LITA was too small to be skeletonized; thus, anastomosis was performed using only milrinone injection. The RITA was also preserved for future stenotic episodes. Angiography performed at ~1 year after surgery showed a good blood flow in both vessels (Figure 4).

Both patients provided written informed consent.

## Discussion

### Examination and Indications of CABG

Guidelines have been developed regarding the indications of CABG for children with KD in Japan. The increasing number of KD cases involving surgery has enabled more detailed evaluations of the indicated pathological conditions, ages, and associated prognoses. In Japan, the indications for pediatric CABG are identical to those for adult cases and include ischemic findings and symptoms. Specifically, CABG is indicated if any of the four following conditions are present: (1) high-grade lesions in the left main trunk, (2) high-grade stenotic lesions in multiple arterial branches, (3) high-grade stenotic lesions in the proximal LAD, and (4) dangerous collateral pathways (3).

In contrast to adults, collateral pathways develop early in infants, and the blood flow is protected to some extent by collateral circulation, even if a stenotic lesion of the coronary artery main trunk progresses. Accordingly, the patient may be asymptomatic during infancy. However, upon reaching school age, the patient's level of activity increases drastically, and new ischemic findings may emerge even without a corresponding change in the degree of coronary artery stenosis. Therefore, a treadmill test or stress/rest myocardial perfusion imaging is useful for determining the indication for CABG. It is important to verify collateral circulation and left heart function by coronary angiography and left ventriculography and to determine the viability of the ischemic region accurately. The remaining coronary artery blood flow is another important determinant during the decision-making process for bypass surgery. In a patient with considerable remaining blood flow, CABG can result in the “string phenomenon,” which involves two competing blood flows and depletion of blood flow from the bypass (4).

### Choice of Bypass Vessel

Kitamura et al. (5) reported the first use of an autologous great saphenous vein in a 4 year-old child with KD. However, this graft resulted in occlusion. In 1985, two adult cases of LITA-LAD anastomoses were performed using the left ITA. Subsequently, a study conducted in 1988 on 12 patients with LITA-LAD anastomoses reported a 100% patency rate, and the authors found that increases in the length and blood vessel diameter of the LITA paralleled the growth of the children (6). Recent literature includes reports of CABG involving the GEA, great saphenous vein, and radial artery bypass; most current cases involve the ITA, which is considered the optimal choice for use in bypass surgery in the majority of cases (7–10). Moreover, the use of the ITA requires anastomosis only with the coronary artery, and the minimal difference in the diameters of these vessels facilitates anastomosis. Moreover, the ITA is unlikely to be affected by KD-associated vasculitis and grows with age.

In the two cases reported in the present study, three of the four vessels were anastomosed to the ITA and only one was anastomosed to the GEA at the distal end of RCA, which did not reach the ITA. The use of GEA is more complicated due to the requirement for laparotomy. Individual variations in the blood vessel diameter and length increase the likelihood of spasms and arteriosclerosis in adulthood.

The radial artery is also available as an alternative; however, the use of this artery has some disadvantages. In particular, it is not pedicled and growth cannot be expected. Some cases involving giant aneurysms of the RCA have been treated with reconstructive procedures rather than CABG, and these procedures have resulted in the establishment of stable blood flow (11).

### Age at Surgery

Statistics regarding the age at surgery in Japan suggest that children as young as 1 year of age have undergone bypass surgeries. The achievement of good post-operative patency rates depends on the facility where the operation is performed. Some facilities would likely perform surgery even in a young child. Previous reports have described the post-operative development of occlusion in some cases. In the two cases reported in the present study, neither patient exhibited findings of ischemia due to the development of collateral pathways. Accordingly, surgery was delayed for several years after considering other factors. The first case also involved aneurysm removal, while the second case involved grafting to the GEA because the RITA could not reach the distal RCA. In the first case, the removal of the giant aneurysm during CABG in infancy may have caused damage to the thin myocardium. Furthermore, in the second case, the narrow GEA diameter and risk of post-operative stenosis were concerning. The surgeon also had had little experience in handling pediatric cases of coronary stenosis due to KD. Therefore, the operation in infancy was postponed until the patient is of school age.

Ultimately, decisions regarding the optimal timing of surgery depend on several factors, including the degree of coronary artery stenosis, condition of collateral circulation, degree of myocardial ischemia, experience of the surgeon, and comprehension on the part of the patient and family.

### Acute Treatment

Both patients developed giant coronary arteries, despite the provision of treatment according to standard guidelines. Although the risk of rupture was high in both cases, this adverse outcome was avoided by imposing an adequate period of complete sedation, ventilator use, and antihypertensive drug therapy to control blood pressure levels. Currently, the acute-phase treatment is largely responsible for the ability of patients with even severe conditions to undergo successful CABG.

### Explanation to the Patient

The prevention of sudden myocardial infarction, a serious complication, also critically depends on the patient's thorough understanding of their illness. The physician should explain the patient's condition adequately to both the parents and the child and should conduct thorough routine outpatient visits and examinations. These practices will enable the patient to remain aware of their illness and understand the need to avoid the types of play that elevate their heart rate.

### Future Treatment and Follow-Up

Both our patients have lived without restrictions on their activities and have only used aspirin since surgery. In the future, it will be important to ensure the prevention of diseases associated with vascular disorders, including arteriosclerosis and diabetes mellitus, by incorporating practices such as daily exercise.

## Conclusion

In this case report, we presented two cases of KD-associated coronary artery stenosis that were treated by CABG after a delay of several years. Both patients developed giant coronary arteries. Rupture was prevented by regulating the blood pressure and heart rate during the acute phase. Ultimately, both patients safely underwent delayed CABG. If the number of such cases increases in the future, the optimal surgical timing and technique for each patient should be examined further.

## Data Availability Statement

The raw data supporting the conclusions of this article will be made available by the authors, without undue reservation, to any qualified researcher.

## Ethics Statement

Written informed consent was obtained from the individual's legal guardian for the publication of any potentially identifiable images or data included in this article.

## Author Contributions

KA wrote the manuscript. MH, HO, and TF followed on these patients. KT, MK, and TS provided language and medical advice. AA, KN, and SK performed surgical treatment and provided advice about surgery. All authors contributed to the article and approved the submitted version.

## Conflict of Interest

The authors declare that the research was conducted in the absence of any commercial or financial relationships that could be construed as a potential conflict of interest.
